# Supplementary material for: Venomix: a simple bioinformatic pipeline for identifying and characterizing toxin gene candidates from transcriptomic data
Source: PeerJ. 2018 Jul 31;6:e5361. doi: 10.7717/peerj.5361 (PMC6074769; doi:10.7717/peerj.5361)
Supplement: Supplemental Information 3 [file peerj-06-5361-s003.gz › FinalOutput_E-20/Snaclec_coagulation_factor_IX@factor_X-binding_protein_subunit_A_1/finaltree.pdf]

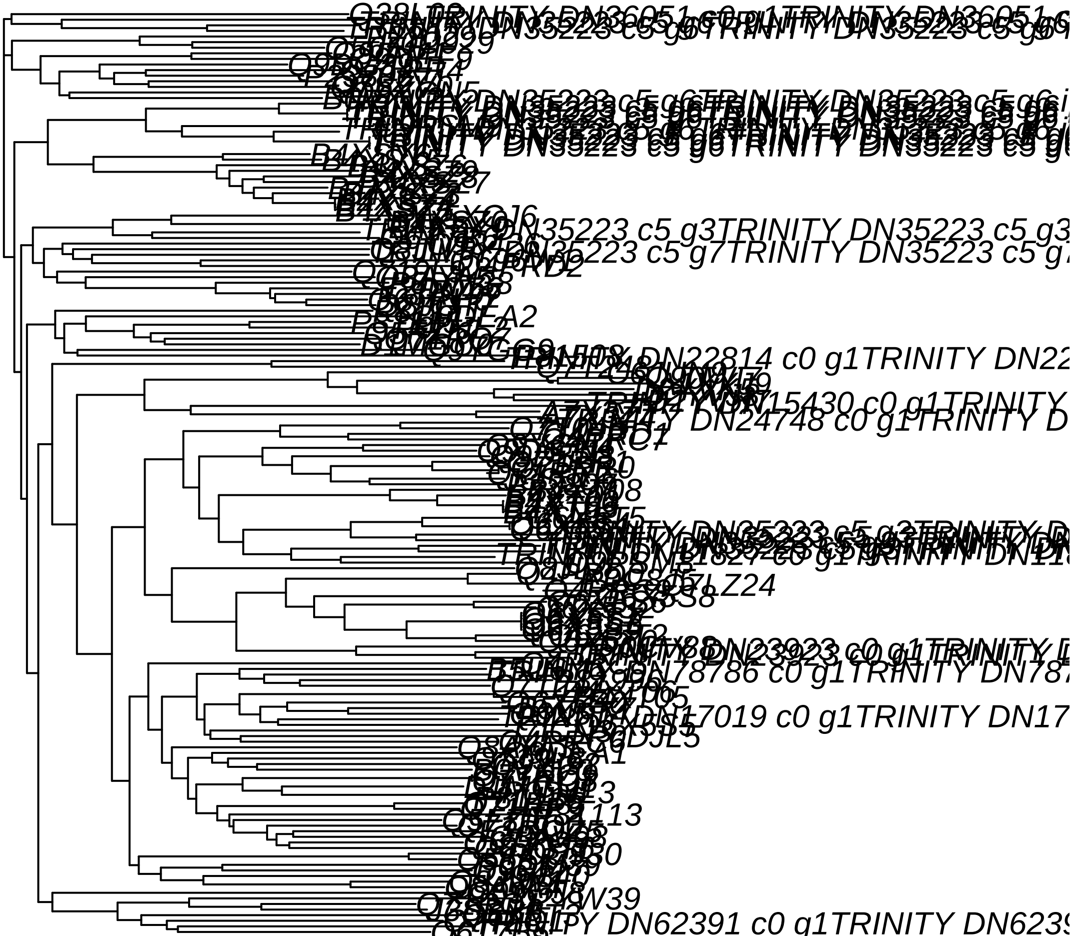
TRINITY DN35223 c5 g3TRINITY DN35223 c5 g3
TRINITY DN35223 c5 g7TRINITY DN35223 c5 g7
TRINITY DN22814 c0 g1TRINITY DN22
TRINITY DN24748 c0 g1TRINITY D
TRINITY DN78786 c0 g1TRINITY DN78
TRINITY DN17019 c0 g1TRINITY DN17
TRINITY DN62391 c0 g1TRINITY DN623
